# Developing attributes and attribute-levels for a discrete choice experiment on micro health insurance in rural Malawi

**DOI:** 10.1186/1472-6963-14-235

**Published:** 2014-05-22

**Authors:** Gilbert Abotisem Abiiro, Gerald Leppert, Grace Bongololo Mbera, Paul J Robyn, Manuela De Allegri

**Affiliations:** 1 Institute of Public Health, Medical Faculty, University of Heidelberg, Heidelberg, Germany; 2 Department of Planning and Management, Faculty of Planning and Land Management, University for Development Studies, Wa, Ghana; 3 Department for Cooperative Studies, University of Cologne, Cologne, Germany; 4 Research for Equity and Community Health Trust (REACH Trust), Lilongwe, Malawi; 5 The World Bank, Washington, DC, USA

**Keywords:** Discrete choice experiment, Attribute and attribute-levels development, Qualitative study, Micro health insurance, Rural communities, Malawi

## Abstract

**Background:**

Discrete choice experiments (DCEs) are attribute-driven experimental techniques used to elicit stakeholders’ preferences to support the design and implementation of policy interventions. The validity of a DCE, therefore, depends on the appropriate specification of the attributes and their levels. There have been recent calls for greater rigor in implementing and reporting on the processes of developing attributes and attribute-levels for discrete choice experiments (DCEs). This paper responds to such calls by carefully reporting a systematic process of developing micro health insurance attributes and attribute-levels for the design of a DCE in rural Malawi.

**Methods:**

Conceptual attributes and attribute-levels were initially derived from a literature review which informed the design of qualitative data collection tools to identify context specific attributes and attribute-levels. Qualitative data was collected in August-September 2012 from 12 focus group discussions with community residents and 8 in-depth interviews with health workers. All participants were selected according to stratified purposive sampling. The material was tape-recorded, fully transcribed, and coded by three researchers to identify context-specific attributes and attribute-levels. Expert opinion was used to scale down the attributes and levels. A pilot study confirmed the appropriateness of the selected attributes and levels for a DCE.

**Results:**

First, a consensus, emerging from an individual level analysis of the qualitative transcripts, identified 10 candidate attributes. Levels were assigned to all attributes based on data from transcripts and knowledge of the Malawian context, derived from literature. Second, through further discussions with experts, four attributes were discarded based on multiple criteria. The 6 remaining attributes were: premium level, unit of enrollment, management structure, health service benefit package, transportation coverage and copayment levels. A final step of revision and piloting confirmed that the retained attributes satisfied the credibility criteria of DCE attributes.

**Conclusion:**

This detailed description makes our attribute development process transparent, and provides the reader with a basis to assess the rigor of this stage of constructing the DCE. This paper contributes empirical evidence to the limited methodological literature on attributes and levels development for DCE, thereby providing further empirical guidance on the matter, specifically within rural communities of low- and middle-income countries.

## Background

There is a growing interest in discrete choice experiments (DCEs) as a means of eliciting stakeholder preferences for healthcare interventions and policy reforms [1-5] to support the prioritization, design and implementation of such interventions [6,7]. DCEs are an attribute-driven quantitative technique used to elicit stated preferences for new products and interventions that are yet to be introduced into the market [8-11].

In DCEs, potential products or interventions are usually described by their characteristics, referred to as attributes, and each attribute is assigned a range of defined dimensions called attribute-levels [12]. The attributes of the interventions and their assigned levels are usually combined using experimental designs to produce a set of hypothetical choice alternatives [12,13]. Respondents are then presented with a sequence of two or more of these competing choice alternatives and are asked to choose which alternative they prefer [1,2]. The attribute-levels determine the utility respondents will attach to a particular characteristic of an intervention, and hence, their choices or preferences [2].

In low- and middle-income countries (LMICs), particularly in Sub-Saharan Africa (SSA), DCEs have been applied within the health sector to elicit job preferences of health workers [14-17], hospital quality assessment [18], priority setting in resource allocation [19], maternal health issues [20,21] and health system reforms [22]. In general, only a few DCEs, none of which are from LMICs, have elicited community preferences for a health insurance product as an intervention in its entirety [23-30]. Specifically, the DCE methodology has not been used to elicit community preferences for micro health insurance (MHI), an innovative health care financing strategy which has received substantial attention in LMICs [31-33].

MHI refers to any voluntary health insurance system that pools funds and risks from members of a community, or a socio-economic organization, to ensure that its members have access to needed care without the risk of financial consequences [32]. MHI schemes are often implemented at the local level, targeting low-income households who work in the informal sector [33]. The premiums paid by MHI members are usually community-rated and the schemes often adopt participatory management approaches, which allow for community involvement in decision making [32,33]. The relevance of applying a DCE to configure micro health insurance products in LMICs emanates from the absence of markets for health insurance products in many such settings. This makes alternative product design and preference elicitation approaches that rely on market-oriented strategies, less feasible in generating timely data to support the design and implementation of MHI interventions in such contexts [2].

As an attribute-based experiment, the validity of a DCE largely depends on the researchers’ ability to appropriately specify attributes and their levels [10]. A misspecification of the attributes and attribute-levels has great negative implications for the design and implementation of DCEs and a risk of producing erroneous DCE results, which can misinform policy implementation. To reduce the likelihood of researcher bias, attribute development has to be rigorous, systematic, and transparently reported [34]. Various methods have been applied to the development of DCE attributes. These include literature reviews, existing conceptual and policy relevant outcome measures, theoretical arguments, expert opinion review, professional recommendations, patient surveys, nominal group ranking techniques and qualitative research methods [2,34,35]. A recent review by Coast et al. [34] casts doubts on whether the process of attribute and attribute-levels development for DCEs is always rigorous, leading to the identification of credible attributes, given the brevity with which it has been reported in existing studies. Acknowledging the limitations of deriving attributes from the literature, Coast et al. [34] argue that qualitative studies are best suited to derive attributes, since they reflect the perspective and experiences of the potential beneficiaries. They insist on the need to accurately describe such qualitative studies and other approaches used in deriving attributes and levels, to allow the reader the possibility of judging the quality of the resulting DCE. There is, however, paucity of such descriptions in the existing literature, in high and low income countries alike [35,36].

Our study aimed at filling this gap by documenting a rigorous process of developing attributes and attribute-levels for the design of a DCE, to elicit community preferences for a potential MHI product in rural Malawi.

## Methods

### Study setting

The study was conducted in the rural districts of Thyolo and Chiradzulu in Southern Malawi. Malawi is a low-income country in SSA with a population of about 15 million [37]. The two districts include approximately 6.7% of the national population [38].

In Malawi, over 60% of all health services are provided by the government in public health facilities; 37% by the Christian Health Association of Malawi (CHAM); and the rest by individual private for-profit health practitioners and traditional healers/herbalists [39]. In principle, healthcare is provided free of charge at point of use in public facilities (tax-funded) and subsidized in CHAM facilities, while private providers rely on user payments [40]. In practice, however, the provision of free healthcare is constrained by constant shortages of drugs and health personnel, and poor infrastructure and equipment, resulting in poor quality, which in turn reflects poor health outcomes [40,41]. A considerable proportion of healthcare is still being financed through direct out-of-pocket payments [40].

The average total healthcare expenditure stands at US $34 per capita, equivalent to 11.7% of Gross Domestic Product (GDP) [42]. There is no nationwide social health insurance scheme, and only very limited coverage of private and employer-based insurance schemes [39]. Due to inadequacies in the current tax-funded system and limited coverage of existing health insurance schemes, private not-for-profit institutions, including microfinance institutions (MFIs), have expressed increasing interest in becoming active agents for the development of MHI, with the aim of increasing social health protection for informal sector workers and rural populations.

The absence of evidence on community preferences for an MHI product, within a predominantly tax-funded healthcare context like Malawi, provided the rationale for our overall DCE study. The intention of the largest MFI in the country, the Malawian Union of Savings and Credit Cooperatives (MUSCCO), to introduce MHI through its Bvumbwe Savings and Credit Cooperative (SACCO), in the Southern Region, provided the policy context for our study.

### Conceptual framework for developing attributes and attribute-levels

There is a growing consensus in the literature that credible attributes and attribute-levels for a DCE must be policy relevant, important to the study population, and consistent with the random utility theoretical foundation of DCE [2,10,34,43]. Policy relevant attributes and attributes-levels are those that adequately reflect the essential dimensions or characteristics of the product, or intervention, that will be evaluated by potential beneficiaries in the DCE [8]. This implies that the identification of such attributes and levels should be guided by appropriate conceptual and theoretical explanatory models and empirical literature on the policy issue. A rigorous literature review on the policy topic can, therefore, lead to the identification of a comprehensive list of conceptual attributes, which can potentially, but not necessarily, be included in a relevant DCE. According to Coast et al. [34], identifying attributes and their levels exclusively on the basis of a literature review may be easier to implement, but may also lead to the non-inclusion of some important attributes. To be included in the DCE, the conceptual attributes must be considered important by the target population, whose preferences will be elicited in the final DCE, and reflect the needs of their local context. This requires a rigorous qualitative study within the local context [34,36]. The attributes and levels derived from such a qualitative study are considered demand-driven [2], reflective of local perspectives, understandable to respondents and thereby, plausible within the study context [34]. Deriving attributes from a qualitative study can, therefore, improve the content validity of a DCE study [10]. A qualitative study is also capable of picking up other context-specific and policy relevant attributes which might not exist in the literature, and hence, can potentially reduce the risk of omitting relevant attributes and attribute-levels. Lastly, the context specific attributes and attribute-levels must be framed in a manner that allows for efficient elicitation and analysis of preferences, according to random utility theory, which is the theoretical foundation of DCE [8]. In this case, DCE attributes (and most particularly levels) must be exhaustive and measurable [2]. The attributes and their levels must be unambiguously framed [27] and appear both cognitively (perceptually) and statistically uncorrelated in the choice sets [44]. Additionally, attributes must be experimentally manipulatable [44], and defined in a manner that gives room for trading between attribute-level alternatives [34]. To ensure these, expert opinion and additional pilot studies within the study area are also recommended [10,34].

Guided by the above conceptual reasoning, we adopted a multi-stage attribute development process, whereby we initially identified policy relevant conceptual attributes from a literature review. We used these conceptual attributes and potential attribute-levels as a basis for designing a qualitative study to identify context-specific attributes, as those deemed directly by respondents to be most important. To scale down the context-specific attributes to a number manageable within a DCE and to ensure that the final attributes and levels conformed to the theoretical postulations of a DCE, we elicited expert opinion and further validated our results through a pilot study.

### Study design

**Figure 1:**
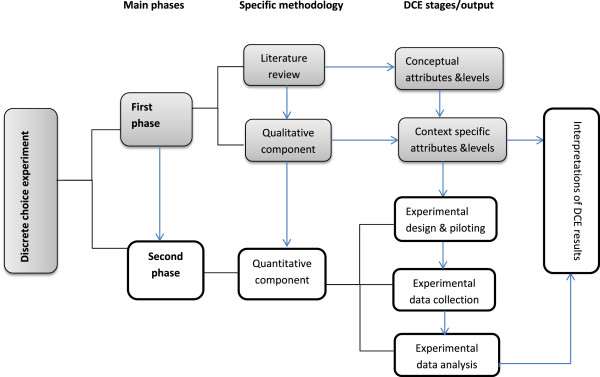
The mixed methods design of the DCE.

The overall DCE study adopts the instrument development variant of an exploratory sequential mixed methods design [45], cognizant of the systematic stage-wise nature of a DCE process [12]. In line with the methodological prescriptions of the exploratory mixed methods design, a qualitative design informed by an initial literature review was used in the first phase of the study, to elicit the relevant attributes and attribute-levels to construct the DCE, and an actual DCE was used to collect and analyze quantitative data in the second phase (see Figure 1 for illustration). As described above, in relation to our conceptual framework, this paper focuses exclusively on the first phase of the study, describing the qualitative component in detail.

### Initial literature review

In line with recent methodological recommendations [4,10,11,34], the attribute development process began with a review aimed at identifying conceptual attributes relevant to an MHI product in the available published literature. PubMed, Google scholar, ScienceDirect, EMBASE and EBSCOhost databases were searched using as first level search terms: discrete choice experiment, conjoint analysis, best worst scaling, preferences elicitation, perceptions, and design features/enrollment/retention/dropout, which were variously combined with second level search terms such as: health insurance, mutual health organizations, health (care) financing, universal (health) coverage and Health Maintenance Organizations (HMOs). Only empirical papers or reviews, policy documents and theoretical/conceptual frameworks on healthcare financing systems and consumer choice behavior, published in English between 1980 and 2013 were considered. For the sake of space, this paper does not discuss the detailed results (e.g.: summaries of single papers), from the literature review, as would be the case in a systematic review, but focuses on the insights from the literature that guided our identification of the conceptual attributes and attribute-levels. In light of the specific circumstances of the Malawian context, a list of conceptual attributes was developed on the basis of four main inputs from the literature:

1. Kutzin’s framework, which defines the four main components of any healthcare financing system as *revenue collection, fund/risk pooling, service purchasing, and service provision*[46], provided a broad framework for attribute identification;

2. Berki & Ashcraft’s framework, which identified direct insurance policy characteristics (*benefit package, premium price and cost-sharing provisions such as deductibles, copayment, coinsurance and benefit ceilings)* and delivery system characteristics (*quality, spatial access, comprehensiveness and continuity*) as the most important features that influence consumer choice when purchasing insurance [47], provided a complementary framework for understanding consumers’ health insurance choice behavior;

3. Existing legislations and policy documents on health insurance in SSA [48-53] and empirical literature on community perceptions about MHI product characteristics, and their relationship to enrolment in MHI in SSA [31,32,54-63], provided evidence on how MHI is currently being implemented within SSA; and

4. Attributes and attribute-levels used in previous DCEs on consumer preferences for health insurance also gave insights into what features of health insurance can potentially be implemented within a DCE [23-30].

Guided by these insights from the literature, three of the authors (GAA, GL and MDA) derived a comprehensive list of conceptual attributes and potential attribute-levels as illustrated in Table 1. The conceptual attributes and their potential levels were used to guide the design of data collection tools for the qualitative component of the study.

**Table 1:** Conceptual attributes and potential levels compiled from literature (adopted to the Malawian context)

***Functions *** [46]	***Based on the frameworks of Kutzin *** [46] ***, Berki and Ashcraft *** [47] ***, health insurance policy documents *** [48,49,50,51,52,53] ***literature on community perceptions on MHI characteristics in SSA *** [31,32,54,55,56,57,58,59,60,61,62,63] ***and attributes and levels defined in previous DCEs*** [23,24,25,26,27,28,29,30]	
	***Policy attribute***	***Plausible levels definition (citations only provided for previous applications in DCEs)***
***Revenue mobilization***	*Who pays the premium*	*Household members, employers*[30]*, Government*
	*Unit of charging premium*	*Individual, household*[26]*, full family*[23,27]
	*Structure of premium*	• *Flat rate*[23,27]
		• *Differential based on: income, employment, age, urban–rural*
	*Premium price (level)*	• *Based on real cost of healthcare*
		•* Based on proposed/existing insurance premiums*[23,29,30]
		• *Based on WTP or qualitative studies*[25-27]
	*Forms of premium payment*	• *Cash*[23-27,29,30]
		• *Material (farm produce) or both*
	*Premium payment mechanisms*	• *Deduction from bank or payroll*[23]*, institutional membership (MFI) account, salary*
		• *Pay through community agents*
		• *Pay directly to insurance office*
	*Premium collection modalities*	• *Pay during wet, dry or all seasons*
		• *Pay weekly, two-weekly*[26]*, monthly*[23]*, yearly*[29]*, installment*
***Fund and risk pooling***	*Unit of enrolment*	*Individuals*[26]*, households, families*[23]*, microfinance institutional or occupational groups*
	*Dependents eligibility*	*None, plus spouse, plus spouse and children*[23]
	*Extent of pooling*	*Family/kin, community, Institutional(MFI) level, district, region, nation*
	*Nature of cross-subsidization*	• *None*
		• *Based on income, employment, risk or geographical location status*
		• *Exemptions for poor and indigents*
***Pooled fund Management and administration***	*Who manages the pooled funds*	• *Names of insurance provider*[26,27]
		• *Community committees,*
		• *Microfinance Institutions,*
		• *NGOs, Health providers, Governmental organization*
	*Quality of customer services*	*Good, bad*[25]
	*Insurance information communication*	*Not provided, weekly, monthly*[26]*, yearly*
	*Enrollment procedure (paper work involved)*	• *No forms to complete, few forms, lots of forms*[26]
***Services purchasing***	*Benefit package*	*Comprehensive, medium, basic packages*
		*Low cost vs. high cost events*
		*Low risk vs. high risk events*
		*Frequently occurring or rare events*
***a. Specific services coverage***	• *Hospitalization due to medical treatment or surgery*[26]	
	• *Medical Consultation (by phone)*[26]	
	• *Pharmaceuticals/drugs prescribed*[25-27]	
	• *Preventive care, wellness and education*[27]	
	• *Vision and hearing care*[26,27]	
	• *Emergency services*[26]	
	• *Mental health services*[26,27]	
	• *Dental services*[26,27]	
	• *Alcohol and substance abuse*[26]	
	• *Treatment abroad or out of town emergency*	
	• *Laboratory, x-ray and imaging*	
	• *Maternal care*	
	• *Consultations of traditional healers*	
	• *Transportation*	
	• *Loss of income when ill*	
	• *Time loss of care giver*	
***b. Cost sharing arrangements***	*Coverage ceiling (maximum liability)*[28]	*benefits within specific facilities, communities, district, national, international*
	*Co-payments levels*	• *None*
		• *Flat rate*[23,30]
		• *A percentage of cost (10%, 25%, 50%)*[26,27]
	*Deductibles*[24,28]	• *Out-of-pocket payment for first visit*
		• *Insurance pays only at a certain quantum of cost*
	*Benefit delivery*	*Cashless and re-imbursement*
***Provision***	*Type of providers*	*Public, private, faith-based or all*
	*Choice of provider (facility)*	*Choose any*[27]*, limited to some, limited to one in the community*[26]*, gatekeeper model*
	*Location of contracted provider*	• *Defined in terms of distance from home or average travelling time to provider*[23,26]
		• *Defined setting: urban, rural*
	*Quality of care*	• *Bad, moderate, good, very good, excellent*[25-27]
	*Reputation of affiliated providers*	*Outstanding, average, below average*[23]
	*Waiting time for care*	*Defined in terms of hours and minutes*[26,29]
	*Opening hours of health facility*	*Only week days, weekends as well, nights and 24 hours*[26]
	*Availability of providers*	*Yes/no*[23]
	*Involvement in treatment decision making*	*Yes/no*[25]

### Identification of context-specific attributes through the qualitative study

#### Study population and sampling

Qualitative data for the development of context-specific attributes and attribute-levels was collected in August/September 2012, using 12 FGDs with community members and 8 key informant interviews with health workers. Community residents (both MFI-members and non-MFI members) were included as potential target clients of the future MHI product in the concerned districts. Health workers were included as key informants because they observe the challenges communities currently face to access care. Triangulating community and providers’ views enhanced the study’s credibility [45]. Since the study deals with a non-sensitive topic, FGDs were deemed appropriate for deriving attributes from community residents, because of the potential of FGDs to yield large amounts of consensual information from a broad range of opinions on a specific topic over a relatively shorter period of time [8]. Moreover, it was relatively easier to organize community residents for FGDs than health workers, who were scattered all over the study area, and hence, could only feasibly be studied through individual interviews [64].

Stratified purposive sampling was used to select both community residents and health workers, and the overall sample size was determined by expected saturation point [64]. For community residents, we applied purposive segmentation to achieve maximal variation, taking into consideration possible diversity in opinions across geographic location, MFI membership status, and sex [64]. First, five traditional authorities (TAs) were purposely sampled to ensure geographical spread across the two districts. Second, one rural community from each TA was selected, relying on evidence of the presence of sufficient MFI members. Third, in each selected community, adult (18+) individuals were selected to participate in one of two FGDs, one including MFI members randomly selected from the MUSCCO-MFI membership list (with sex being held as sole purposive sampling criteria) and one including non-MFI members sampled from the community. Men and women were separated into different groups. Though women are generally more involved as members in the local MFI than men, a total of 6 women’s groups and 6 men’s groups were formed. Community leaders assisted the data collection team (GAA and two research assistants) to recruit 8 to 12 participants for each FGD.

Health workers from health facilities in the concerned areas were purposely selected to represent public, faith-based (CHAM), and private-for-profit providers. In each sampled facility, the most experienced (senior) health worker was selected for interview, resulting in a sample where almost all the 8 health workers were facility heads.

### Data collection

The list of conceptual attributes (Table 1), developed on the basis of the existing literature, served as the basis for the development of one single interview/discussion guide used to conduct all FGDs (see Additional file 1). The guide was adjusted to conduct the interviews with health workers. The use of a guide was necessitated by the limited familiarity of the concept of MHI among the study participants and, hence, a need to provide moderators/interviewers with a common instrument, as a means of ensuring uniformity in the topics to be discussed across all groups. The interview/FGD guide was semi-structured around a list of open ended questions, including adequate probes. The guide was comprised of two main sections. The first section aimed at deriving attributes, and hence, it contained broad questions on: how participants experience the healthcare system and provision gaps; how participants would like an MHI scheme to be designed; the product attributes they would value as important when deciding whether or not to join; and the motivations for their responses. Respondents were initially allowed to openly discuss the above topics. Afterwards, to ascertain their importance, moderators probed for MHI characteristics that were identified in the literature, but not spontaneously mentioned by the respondents during the FGDs. The second section aimed at deriving specific attribute-levels. Hence, using the comprehensive list of potentially relevant attributes as a guide (Table 1), participants were asked to identify probable options for each attribute.

All FGDs were conducted in the local language (Chichewa) by the two research assistants; one serving as facilitator and one as note-taker. Before the discussion, the facilitator provided respondents with a detailed explanation of the MHI concept, using as illustrations locally appropriate expressions and images (see Additional file 1). All FGDs were tape-recorded, transcribed, and translated into English for analysis. FGDs lasted, on average, 2 hours. All FGDs were conducted in secured, enclosed places, such as schools or churches, free from external distraction.

All interviews with health workers were conducted in English, directly by GAA, tape-recorded, and later transcribed. Each interview lasted between 45 minutes and one hour.

### Ethical approval

Ethical approval for the study was obtained from the Ethical Committee of the Faculty of Medicine of the University of Heidelberg in Germany and from the National Health Science Research Committee (NHSRC) in Malawi. Before data collection took place, permission was also obtained from the district commissioners, the district medical officers, and the local authorities of the concerned communities. Written informed consent was obtained from all study participants. All sampled respondents consented to and participated in the study. To enhance confidentiality, all FGD participants were encouraged not to discuss each other’s opinions outside the FGD setting. Also, to make it less possible for respondents’ opinions to be easily linked to their personal identities, names of respondents were not recorded. We have adhered to the RATS guidelines for qualitative research modified for BioMed Central instructions to authors.

### Data analysis

To ensure inter-researcher reliability, analysis began with an independent reading, coding, and categorizing of the qualitative transcripts by three different analysts [64]. GAA analyzed the entire material using the computer assisted qualitative data analysis software NVivo (version 9). He relied on a pre-established coding scheme developed on the basis of the FGD/interview guide and the conceptual attributes identified in the literature, but allowed for new codes and categories to emerge as he proceeded through the reading. MDA and GBM manually analyzed two-thirds of the material. They approached the material inductively, letting codes and categories emerge as they worked their way through the transcripts. At a later stage, the three analysts compared the results of their analysis to obtain one single list of all elements identified by community, and by providers, as attributes and relevant levels. Discrepancies in interpretation were reconciled by returning to the text, “questioning” the transcribed material to identify which elements really reflected an attribute and which ones did not.

### Expert opinion

This step was aimed at reducing the attributes to a number manageable within a DCE, by discussing the list of context-specific attributes derived from the qualitative analysis with two sets of “informed” people, purposively selected based on their experience with the DCE methodology. These discussions served the purpose of ensuring that the selected attributes were consistent with the methodological postulations of DCE. The list was also discussed in a group setting with five purposively selected researchers familiar with Malawi and with MHI. This was to further ensure that the selected constructs not only appeared credible and realistic in the Malawian context, but also adequate to answer important pending research questions on community preferences for MHI in SSA.

### Self-reflection and additional insights from a pilot study

In this stage, the research team gathered to revise the list of attributes in light of the feedback received during step two. This last step allowed for one last collective credibility and reality check on the list of retained attributes and levels. Using the list of attribute and levels retained at this stage, a quantitative DCE pilot study was designed and administered to 49 respondents. The aim was to derive the parameters for the actual DCE design, to test other components of the DCE design and to assess the clarity of the wording, as well as appropriateness of defined levels and local translations, and comprehensibility of attributes and levels within the choice sets [10]. The last element is of specific relevance to the concepts and experiences described in this paper. The interviewers working on the pilot were specifically instructed to observe and document the respondents’ reactions and comments on the attributes and attribute-levels used during the pilot. Their observations were discussed within the framework of an FGD, bringing together all the interviewers.

## Results

### Qualitative analysis of the transcribed material and initial attribute identification

In total, 127 residents participated in the FGDs. These included: 64 from Thyolo and 63 from Chiradzulu districts; 64 males and 63 females; and 61 SACCO and 66 non-SACCO members. The eight health workers were comprised of two medical doctors, one from a CHAM hospital and the other from a public district hospital; two nurses/midwives, one from a CHAM hospital and the other a public district hospital; two medical assistants/clinicians from the two public clinics; and a clinician and a paramedic from the two private health centers. The health workers from the private sector and the medical doctor from the CHAM facility had previously worked in the public sector, while two of the public sector workers had also previously worked in CHAM facilities. The health workers who participated in the study had experience within the Malawian health system ranging from 2 to 48 years.

Table 2 displays the complete list of all attributes and attribute-levels identified by consensus among the three analysts during the initial triangulation process. They include: premium level, premium collection modalities, premium structure, unit of enrolment, geographical level of pooling, management structure, health services benefit package, transportation coverage, copayment levels, and provider network (i.e. the type of health facilities to be contracted by the MHI). To give voice to the respondents’ views on attributes and their levels, direct quotations, poignantly selected, from the qualitative transcripts are included in Table 2.

**Table 2:** Derivation of final list of DCE attributes and plausible levels (ordered from most preferred to least)

**Attribute label**	**Lay terminology**	**Key quotations from qualitative data (mostly FGDs)**	**Labels of plausible levels**	**Final inclusion**
Unit of enrollment	How many family members will benefit from enrollment into the MHI scheme	• “*If everybody in my family will benefit from this basket… it will be a good idea, … but if I am the only person to benefit since I will be the one contributing into the basket, then it is not a good idea since I will still be paying hospital bills for my dependents” (Non-SACCO men)*	• *Entire extended family*	Yes
		• “*The head of the family should pay on behalf of the whole family” (SACCO women)*	• *Core nuclear family*	
		• “*If it offers a package covering them and their children, they will be more than happy to go for it” (Health worker at district hospital)*	• *Individual*	
Management	The managers of the common basket	• “*Sometimes, just seeing the leaders who are managing this thing can make one to join or not” (SACCO men)*	• *Community committee*	Yes
		• “*There should be an elected committee to run the basket and trusted people” (SACCO women)*	• *An external NGO*	
		• “*I will be happy if this basket is managed by the community for easy monitoring and accessibility” (Non-SACCO men)*	• *Bvumbwe SACCO*	
		• “*If the basket can be managed by the NGOs it can be a good thing because if it is managed by people of this community…. if they buy chicken with their own money, people might think that they are misusing the money from the basket” (Non-SACCO men)*		
		• “*I think the SACCCO can manage it but there should be a committee from the community …. linked to the SACCO, if it is managed by only SACCO there will be no trust” (SACCO-Men)*		
Health service benefit package	The health services that the MHI will pay for	• “*There are some drugs which cannot be found at public hospitals except private hospitals, so this basket should cover these situations” (non-SACCO men).*	• *Comprehensive: Drugs, lab test/ x-ray, and surgical operations*	Yes
		• “(It should cover) *x-ray and drugs, no more things (services) because we can’t manage to pay” (Non-SACCO men)*	• *Medium: Drugs, lab tests/x-rays*	
		• “*We have all agreed that medicine should be included in this basket” (SACCO women).*	• *Basic: Drugs only*	
		• “*They have to be sure that once they are putting money into this insurance, they are going to be covered properly” (health worker at private clinic)*		
Copayment	The proportion of health service bill that a MHI member is expected to pay out-of-pocket	• “*The basket should be assisting with half of the bill not the whole bill” (SACCO women)*	• *None*	Yes
		• “*25% (*from the patient*) is fair ….. because we should think of others who will also need the basket” (non-SACCO men)*	• *25% (quarter)*	
		• *“It can happen that you are sick but you don’t have a single coin … the committee is telling you, you will only get 50% of your charge from the basket, the other half will be paid by yourself…it will mean the basket will be of no use” (Non-SACCO men )*	• *50% (half)*	
Transport	Transport	• “*I will join …… if I fall sick and this basket will cover transport to the hospital“ (SACCO Men).*	• *Always from home to the health facility any time sic*	Yes
		• “*Private hospitals are very far from here so we need transport from this community to these private hospitals” (SACCO women)*		
		• “*Transport, because we have problems mainly in times of referral to Thyolo hospital” (district hospital) (Non-SACCO Men)*	• *Only during referral and emergencies*	
		• “*If they package involves offering transport to people from where ever they are to here, they will be more than happy to join” (health worker in public health center)*	• *none*	
Premium per person per month	Membership contributions	• “*If the contributions will be unaffordable then I cannot join” (SACCO women)*	• *MWK100*	Yes
		• *“We will manage MWK100 per month, if they charge more than that; people will not be able to pay” (Non-SACCO-women)*	• *MWK300*	
		• *”We should agree on MWK500 per month” (Non-SACCO men)*	• *MWK500*	
		• “*The amount of money to be contributed whether is it monthly or how often” (health worker, private clinic)*		
Premium payment modalities	Frequency of premium contribution	• “*Here, most of us find money on a seasonal basis, so I think it would be ideal if we contribute at the beginning of each and every year” (SACCO women)*	• *Once-off annual payment*	No
			• *Monthly payment*	
		• “*Monthly contribution will help to have more money in the basket than annually” (non-SACCO men).*		
Provider network	Contracted healthcare facilities for service provision by the MHI	• “*When a person falls sick and goes to private hospital, he should use the money from the basket to settle the bills because there is a difference between public and private hospitals in terms of treatment“ (non-Sacco men)*	• *Private –for-profit*	No
			• *Faith-based (CHAM )facilities*	
		• “*They will like to go to private facilities” (Health worker, public facility)*	• *Public health facilities*	
Pooling level	Extent of geographical pooling	• “*Each and every village has to have its own basket” (non-SACCO Women)*	• *Community level*	No
		• “*I cannot be happy with district level” (non-SACCO Men) ”… there will be no trust and some will benefit from it while others will not benefit ……. unless it is at district level and managed by NGOs” (Non SACCO men)*	• *Traditional Authority*	
			• *District*	
Premium structure	Extent of dependency of contributions on earnings	• “*It should be one figure because everyone whether one earns more or less can fall sick so it should be one figure” (SACCO Men).*	• *Flat rate contributions*	No
			• *Contributions based on earnings*	

Attribute-levels were extracted directly from the transcripts, as illustrated by the relevant citations (Table 2). Only the three most relevant attribute-levels were defined for each attribute, to ensure design simplicity and easy recognition by respondents [10]. Only two attributes, premium level and health service benefit package, deserve further explanation.

In line with existing methodological recommendations [44], levels for the premium were set to reflect the complete range of amounts agreed upon in the FGDs. The assumption was that the later DCE should elicit a realistic marginal willingness-to-pay (WTP) value, rather than reflecting the actual cost of the MHI product (which needs to be subsidized in any case). Levels for the health service benefit package were derived by combining the single services frequently mentioned during the FGDs (drugs, laboratory tests, surgery) into meaningful incremental clusters. FGD participants mainly argued that the benefit package should only include services for which they identified a current lack of effective coverage through public provision. Some services were mentioned as important, such as maternity care, but recognized as adequately provided by governmental facilities. These were excluded from the benefit package, with the rationale that MHI will be set to fill gaps in coverage and not to substitute existing public service provision [31].

“*Maternal care should not be in the basket because; such complications are in the hands of the public hospitals. Any time there are such cases, the hospital calls the ambulance to assist by taking the patient to the district hospital, so no need for antenatal mothers to be included in the basket*” (Non-SACCO men).

### Step two: Selecting relevant attributes in the light of experts’ feedback

The iterative process of discussion with additional scientists led to the retention of 6 out of the initial 10 attributes identified in the qualitative material. The discussion was oriented to limit the number of attributes to between 4 and 8, in order to later allow the DCE to contain a manageable number of alternatives, that would not overwhelm respondents [1]. The last column of Table 2 indicates whether an attribute identified during step one was retained in step two. The discussion with additional scientists also allowed the team to redefine the language used to describe both the attributes and the relevant levels, often requiring a return to the original text to identify the specific terminology used by the community. This was meant to ensure consistency with the Malawian context.

Multiple criteria guided the choice of attributes to be dropped. First, attributes and/or levels that overarched/overlapped other attributes were discarded in order to avoid cognitive inter-attribute correlation [44]. For instance, pooling levels overlapped management structure since both had a geographical dimension; or preferences for premium collection modalities will depend on the premium amount – see Table 2. Second, attributes for which clear preference was established in the FGDs for certain levels were dropped to avoid dominance. There was clear preference for: private-for-profit and CHAM facilities (as a proxy for quality of care); fixed rate premium payments; and pooling at the community level. Finally, attributes were dropped if, in the FGDs, they had been identified as elements of secondary importance, such as pooling level, which entered the discussions only after persistent probing. However, fixed levels were defined for all discarded attributes as part of the introduction to the choice exercise. This reduces the tendency of respondents inferring levels for such attributes which can potentially introduce unobservable biases into the final DCE estimates [8].

### Step three: Final attribute selection and revision in the light of results from the pilot study

After the reduction and revision process of step two had taken place, the research team once again discussed the relevance of the selected items, their feasibility, and comprehensibility in the local context. Only minor changes in terminology were applied to the attribute levels. The core team agreed that all attributes and levels selected during step two satisfied the essential characteristics of a DCE attribute, i.e., they reflected the characteristics of an MHI product; were deemed important by the community; were understandable; and mutually exclusive in nature [34], and retained them for the final DCE.

The analysis of the final DCE pilot results (run primarily to generate prior parameters for the DCE design) confirmed the theoretical validity of the defined attributes and levels, since all had the expected signs, though few were significant; probably due to insufficient sample size (*n* = 49). The FGD with the four research assistants who administered the pilot study revealed that respondents did not raise any major concerns relating to the appropriateness of the defined attributes and levels. Only a few minor revisions were made to the local translations of the attributes and attribute-levels. The pilot, therefore, enabled the confirmation and validation of the final framing of the attributes and attribute-levels, as illustrated in Table 2. The pilot also indicated that participants had no cognitive difficulties in identifying and understanding the attributes and their levels. The interviewers argued that this result was achieved due to the fact that attributes and their levels were illustrated to respondents using context-specific pictures.

## Discussion

This paper contributes to the literature on DCE attribute and attribute-level development [35,36], by explicitly reporting on the systematic process of deriving attributes and attribute-levels for a DCE to elicit preferences for an MHI product in rural Malawi. This study built on the initial identification of conceptual attributes from the literature to develop a detailed interview/discussion guide used to gather primary qualitative data at the community level in a systematic manner. A rigorous analytical process, characterized by three sequential steps, allowed for the identification of relevant attributes and their levels.

Basing the interview guide on the results of the initial literature review, spanning from conceptual to applied studies, allowed the research team to identify a preliminary broad series of attributes and attribute-levels that reflected all possible important, and hence policy relevant, components of an MHI product. Directly engaging with communities and health workers allowed the research team to work through this initial conceptual and very comprehensive list, to select context-specific attributes that were understandable and important in the eyes of the potential beneficiaries of the insurance scheme [34]. The citations that accompany the attributes and the relevant levels, in Table 2, offer a clear indication of how decisions on attribute and levels selection were rooted in the voices of the potential beneficiaries. The qualitative process also provided a clear understanding of the likely order of preferences (most to least preferred) for the various attributes levels. This enabled the design of DCE packages to actually compel respondents to make trade-offs in their choices [34].

This initial qualitative phase, and the attribute validation pilot study, also offered the research team the added benefit of framing the final DCE choice sets in line with local concepts and terminology. This has the potential of maximizing response efficiency in our DCE, thereby enhancing the content validity of the study [1,2,10]. The qualitative process also offered the opportunity to identify and exclude attributes and levels that are potentially dominant, less tradable, less important, and perceptually correlated, from the choice sets, in order to fully satisfy the credibility criteria of DCE attributes and levels [2,34,44].

Four of the final attributes derived - premium level, management structure, health service benefit package, and copayment levels - reflect what had been used in prior DCEs exploring preferences for health insurance products in high income settings [23-30]. However, unit of enrollment, as defined in our study, and transportation coverage might not have been included had we relied only on the literature review. This supports the relevance of conducting qualitative studies to enhance the contextual appropriateness of DCE attributes and levels development [8,10,34].

Coast et al. [34] argue that an iterative constant comparative approach to data collection and analysis is generally preferable for attribute derivation, because of its ability to constantly adopt the research questions in the light of emerging findings. Within the particular context of our study, however, such an approach would have not been feasible for a number of reasons. Geographical distance between the research team and the concerned communities, as well as obvious language barriers, made it impossible for the researchers themselves to engage in a constant iterative process during all phases of data collection and analysis. Feasibility concerns dictated the organization of the data collection and analysis phases. An iterative constant comparative approach, however, was applied within an analytical process, also supported by the rigorous application of the triangulation principle. Had the analysis revealed that saturation had not been reached, however, the research team would have returned to the field to gather more data [64]. The experience reported in this paper indicates that in the event of feasibility constraints of adopting a fully iterative approach to data collection and analysis, other rigorous qualitative approaches can yield equally relevant results for the development of credible attributes and attribute-levels.

Most prior qualitative studies aimed at deriving attributes were conducted among people who had experienced the phenomenon under consideration [26,36]. The limited exposure of our participants to health insurance schemes represented a major challenge. This compelled us to seek out innovative ways of explaining the concept of MHI using appropriate local images and diagrams, and adjusting MHI social marketing concepts and illustrations from other SSA settings to fit local socio-cultural constructs (see Additional file 1) [31]. The concern that the original framing of the FGDs might have influenced the participants’ responses, however, was dissipated by the fact that findings from the individual interviews with health providers largely confirmed findings from the FGDs. Since MHI represents one of the many financing options being discussed at a higher policy level, health workers, unlike communities, had already been exposed to the concept at the time of the study and could not have been influenced by our framing.

Based on the experience of this team, the analysis of the data generated from this type of qualitative study is often challenging. This is because while qualitative studies often generate large volumes of data, attribute development requires only little information on what community members see as important attributes and levels. Given the amount of time and resources that are often spent collecting data, researchers could develop the impression that not all the data, such as the detailed illuminations and explanations of points provided by the study subjects during the FGDs and interviews, have been adequately used. Moreover, it is a common tradition in public health that scholars cherish results that are statistically representative of the study subjects [34]. A qualitative study is not always able to generate this “representative” information, since such studies aim at illuminating complexities and revealing similarities and differences, instead of counting opinions [64]. Selecting attributes and levels based only on qualitative studies, as in our case, could attract criticisms from quantitatively biased researchers, who may argue that at least basic quantitative tools, such as best-worst scaling and nominal group ranking techniques, should be included within the qualitative approach in selecting attributes [35]. Therefore, it could be a good idea to use such simple quantitative tools, after the rigorous qualitative exercise, to support the scaling down of the potentially numerous attributes and levels, that will be generated from the qualitative study, to a number manageable within the DCE. In this case, it must still be guaranteed that the final attributes and levels selected are capable of being used within the DCE, and this would still require qualitative reasoning and deductions.

## Conclusion

This study complements existing literature on DCE attribute development, by providing a detailed account of the scrupulous application of recently recommended approaches to attribute and attribute-level development and reporting [10,34]. Our applied approach is based on the adoption of literature as the starting point, to inform comprehensive field qualitative data collection, followed by a rigorous analytical approach, supported by a series of triangulation and validation exercises. As such, our study provides additional empirical guidance on the methodological processes of developing attributes and attribute-levels for DCEs specifically within rural communities in LMICs. A transparent description of the attribute development process of DCEs provides useful grounds for the assessment of the rigor of this process in DCEs [34], and hence, should receive more attention in future DCE studies. The potential of DCEs to support the design and implementation of interventions, therefore, largely depend on the credibility of the attributes and attribute-levels used in the experimental design.

## Abbreviations

CHAM: Christian Health Association of Malawi; DCE: Discrete choice experiment; FGD: Focus Group Discussion; GDP: Gross Domestic Product; HMO: Health Maintenance Organization; LMICs: Low – and Middle-income countries; MFI: Micro finance Institution; MHI: Micro Health Insurance; NHSRC: National Health Science Research Committee; MUSCCO: Malawi Union of Savings and Credit Cooperatives; SACCO: Savings and Credit Cooperatives; SSA: Sub-Saharan Africa; TAs: Traditional Authorities; US: United States; WTP: Willingness to pay.

## Competing interests

The authors declare that they have no competing interests.

## Authors’ contributions

GAA, GL and MDA conceptualized and designed the study and its data collection tools. GBM supported the design of the data collection tools. GAA administered and transcribed the interviews with health care workers, and supervised the data collection. GBM supervised the transcription of the FGDs. All authors participated in the data analysis. GAA wrote the first draft of the manuscript. GBM, PJR, GL and MDA revised the draft. All authors read and approved the final manuscript.

## Pre-publication history

The pre-publication history for this paper can be accessed here:

http://www.biomedcentral.com/1472-6963/14/235/prepub

## Supplementary Material

Additional file 1 Data collection instruments. Click here for file
