# Supplementary material for: Developing attributes and attribute-levels for a discrete choice experiment on micro health insurance in rural Malawi
Source: BMC Health Serv Res. 2014 May 22;14:235. doi: 10.1186/1472-6963-14-235 (PMC4032866; doi:10.1186/1472-6963-14-235)
Supplement: Additional file 1 — Data collection instruments. [file 1472-6963-14-235-S1.doc]

**Appendix**

**1: A Focus Group Discussion Guide: Exploring attributes and attributes levels of MHI from community residents in Malawi**

***(Note: For attributes and levels derivation, go to part C and Part D)***

**Part A: Basic information about FGD (to be printed out and filled by FGD facilitators)**

| Focus Group Discussion no. | |
| --- | --- |
| Starting time |  |
| Name of district |  |
| Location /Setting |  |
| Name of community |  |
| Sex of group |  |
| Type of group |  |
| Closest health facility (ies) (name and type) |  |
| Number of participants |  |
| Name of FGD facilitator |  |
| Closing time |  |

**Introduction:** I am (moderator’s name) and my colleague is called (note-taker’s name). The two of us are working for REACH TRUST, a research organization from Lilongwe. The third person is………. He is from Ghana, but currently working at the University of Heidelberg in Germany. REACH TRUST and the University of Heidelberg are jointly conducting this study in collaboration with a local organization, the Bvumbwe SACCO. This discussion that we wish to have with you today is part of a bigger study that is aimed at assessing the potentials of a proposed health protection program called health insurance in meeting the health care needs of people within this community or district. Some organizations are planning to introduce it within this district and we would like to find out your opinion about it and how it should be designed to reflect the way you will like it to be. We are interested in gathering your views on such potential programs they wish to launch to improve people’s livelihood in the area. Specifically, we will discuss the following issues with you.

- Your health care needs and the problems you face in accessing the health care you need
- Your understanding of and experience with health insurance in general
- We will introduce to you the type of health insurance (health protection plan) that is being proposed and we will like to get your opinion on the elements that you will consider important in designing such a system
- We will also like to know and discuss with you the important elements that will influence your willingness to join/accept such a system if it is present here in your community or district. We will discuss topics including the contributions that you will be expected to make to the system, the benefits that you will expect to get from it and how you would like the program to be managed.
- We will also specifically identify and discuss the kind of health services you will like to be included in the benefit package of this system

We selected you for this discussion because people like you and your households are targeted for enrollment into this proposed program. Therefore, as people who will be making decisions on joining this program, it is important that your views are taken into consideration when designing the program. We have not been sent by those organizations that are planning to introduce this program to hold this discussion with you for them. We are independent researchers. However, we will inform those organizations of the results that we will obtain from this discussion in a manner that nobody can link the information to any single one of you as an individual. If they take your opinions into consideration, they will be well informed on the important elements they should consider in designing the program to meet your needs. Our discussion will last for not more than 3 hours.

We have obtained ethical clearance for this study from the University of Heidelberg and the Malawi National Health Sciences Research Committee.

Statement of informed consent: *(facilitator to read out from written informed consent form and obtain signature/ thumb print)*

***Start tape recording if consent is granted: (Facilitator to switch recorder on)***

**Part A: Community health care problems and needs**

1. As members of this community, what are your (main) problems in accessing all the health care you need?

Probes: (probe only if the issue is not mentioned):

- Cost associated with seeking health care
- Availability of health providers and medical products
- Quality of health care (waiting times, perceived quality of drugs, attitude of health workers)

1. Do you think anything could be done to help you overcome such problems? If so, what? And by whom?
2. **Community awareness, understanding and experience with health insurance**

We would like to explore your experience with health insurance schemes in general.

- 1. Have you ever heard about health insurance?
- Probe further using terms like medical schemes, sickness funds, mutual health organization.
- If nobody has ever heard about the above, probe further by saying what about MASM?
- (If nobody in the group has heard about health insurance, go to 1.6.) but probe about funeral insurance.
  1. How did you first hear about health insurance?
  2. What do you think is the function of health insurance?
  3. Are you members of any health insurance scheme?
- Probe for the names of the insurance schemes, types of health insurance schemes- employer-based, private etc
  1. Can you tell us what you know about (these) health insurance systems?

Probes:

- What kind of people are members of these schemes
- Premium levels and how it is paid
- health services covered (benefit package)
- proportions of cost paid by insurance
- health providers who render services to (insured) clients
  1. For those who are not on health insurance, how do you normally pay for health care when you fall sick?
- Probes: Out of pocket, access free, do not access health care at all-why?.
- For each payment mechanism probe further for; at what kind of facility-Public, Private or CHAM, traditional healers
  1. What kind of health services do you normally have to pay out of your pocket for and at what type of health facility? Why? Use list of services in Table 1 as probes:
  2. Are there any health services you think you need but they are not available at all or that are available but you cannot access them? Why?
- Probe for names of services and why they are not available or not accessible.
- Probe whether respondents know where those services that are not available to them can be obtained

**Part B: Introduction to insurance, health insurance and MHI**

**Facilitator:** *Read out the following slowly and in a demonstrative manner to participants*

Insurance in general is like you going to buy some warm jacket, even when the weather is not cold so that any time the weather gets cold, you will wear it to protect yourself. The reason is that at the time the weather gets cold, you may either not have money to buy a good jacket or the jacket may be so expensive that you may have to sell your valuable assets in order to buy it or else you will not be protected against the cold weather.

However, it must be noted that it is possible that a single person might not be able to buy this Jacket alone. A group of people may therefore come together to contribute money to buy a number of jackets so that when one person feels cold, one jacket will be available for him/her. Since the single person could not have bought a jacket alone, but pooling money together, there are enough jackets to cover all cold people. The idea of pooling is very important here. However, the problem in this scenario is that it is possible that all the people can feel cold at the same time when the weather suddenly gets cold and may all need to wear a jacket at the same time. The Jackets bought by the group may therefore not be enough for all the people.

Health insurance is like this jacket example. Feeling cold and requiring to wear a jacket is like falling sick and requiring medical treatment. Getting the jacket to wear is like getting a successful treatment for your sickness without paying for the treatment at the time you are sick. But, you will agree with me that in terms of falling sick it is not likely that the whole community or group will fall sick at the same time and thereby needing health treatment (wearing of the Jacket) at the same time.

Health insurance is therefore a system where a group of people (both sick and healthy, poor and rich) come together and agree to be putting money regularly into a single basket so that when one of them falls sick, his/her treatment cost is paid from this common basket. The treatment costs can be in fact much higher than the actual amount that the person has put in the basket and still the cost will be paid from the money that has accumulated in the basket. The rational is also that, at the time you fall sick, you may not have enough money to meet the high cost of a good treatment. This may prevent you from obtaining a good treatment for your sickness or compel you to sell your valuables to pay for the treatment. If you contribute into the basket and stay healthy, you are not given a refund of the money you put into the basket (fund). This is because it will be used to support the treatment of those who will fall sick within the group. In effect, the entire community or group is sharing risk of health care and ensuring that each member has access to good health care.

How people contribute into this common basket (insurance fund), who benefits from this basket, what health care services should be bought from this basket, how the relevant health care services will be provided and how the money will be managed, are all elements to be decided when designing the system. Those who will benefit from the system are normally expected to make an input into the design of those elements. So, we want you to contribute to this decision making process. Hence, these are among the several things we will like to discuss with you today.

*(If some members indicate awareness and experience with health insurance, facilitator should add this:* Those schemes that currently exist in Malawi and which some of you are already aware of or are members of (*restate names of schemes*) are either private health insurance schemes or employer specific schemes. The private health insurance schemes are like business organizations and hence aimed at making profit).

The type of health insurance that has currently been proposed by civil society organization and we are here to discuss with you about, refers to a system that will be designed based on the interest of the people it intends to cover and hence will not be for profit. This is the reason why it is important to obtain your opinions on how you will like it to be designed. It is called micro-health insurance (MHI). From now onwards, we will always use this name MHI to refer to the insurance program we are here to discuss with you about.

Within a MHI, everyone in a group (for example a MFI group (*for SACCO members, say like the SACCO)* or a community (for example name of community) or within the entire district (name of district), who is willing, pays money regularly into the common basket and the money accumulated in this basket is used to pay for health services for each member (sometimes, including his household members) when ill.

MHI systems exist in a number of communities in countries such as Ghana, Burkina Faso, Cameroon and some other countries in the world. In one district in Cameroon for instance, the people have established a MHI fund (common basket) which is managed by elected representatives of the community. To become a member of the MHI, people pay a registration fee and an annual contribution (premium) into the common basket to cover their entire household members (usually only 4 people). Each member is given a membership identity card and any time the member falls sick, s/he goes to the hospital with his/her card for treatment and the insurance fund pays 75% of the total cost of the treatment [1]. In Ghana, a similar system exists, but in the Ghanaian system the insurance pays for all the cost of the services provided. However, in Ghana, each member’s contribution covers only him/herself alone but not the entire household as is the case in Cameroon. In Ghana also, children and the very poor in the community are not expected to contribute into the fund but they also enjoy the health services free of charge and the insurance pays for them. In both the Ghanaian and Cameroon examples, every member is expected to pay his/her contributions (premium) yearly irrespective of whether s/he benefited from the scheme in the previous year or not.

It must be noted that unlike in countries such as Ghana where MHI has been encouraged by the government as a way of replacing the already existing system of financing health care, in most cases, MHI is promoted by organizations and companies other than the government to supplement what the government is already doing to provide health services to the people. As we indicated earlier, this MHI we want to discuss with you has been proposed by development oriented organizations but not the government. The introduction of this MHI will therefore not stop the government from continuing with what it is already doing in providing health care services to you. It is supposed to be an additional support to those who will be part of the insurance to access good health care. Again this is a reason why it is very important for you to make your opinions known to policy makers on how it should be designed to reflect the way you will like it to be.

*2. Ask participants the following questions to test their understanding of the basic principles of MHI and their general opinions about MHI*

2.1. Do you now feel that you understand what a MHI is?

- *(Facilitator will make sure that participants understand the key principles of MHI: Regular and advance payment of money into a common basket that will be used to buy health services for the contributors)*

2.2. Would you consider a MHI to be useful for you and your community? Why?

- Probe for how it can help them overcome some of the problems in accessing health care

2.3. Would you like such a system to be introduced now into your community? Why?

2.4. Would you be willing to contribute towards such a system? Why?

**Part C: Deriving attributes of MHI (General questions)**

***3.1. Preamble:*** Based on the way we have just explained MHI to you and what you know about this community/district, if you are asked to give your opinion on how you will like a MHI system to be set-up within your district.

3.1.1. What specific elements come to your mind spontaneously that you will consider very important in designing such a MHI. (For each element mentioned, probe for reasons by asking why?)

3.3.2. Probe for other elements not mentioned in the open discussion by using Table 1 as guide)

3.2. Assuming a MHI is present in your community (name) or district (Chiradzulu or Thyolo), which elements of this MHI will you consider important in taking a decision to join it or not? (Facilitator should ask for reasons behind elements elicited by probing)?

- Probes***: For each element that is mentioned, the facilitator will probe for reasons by asking:*** Why will *(state element as mentioned by participants)* be important to you in taking a decision to join or not to join the MHI?

**Part D: specific questions (further exploration of attributes and derivation of attribute levels**

***Introduction:*** *In the rest of the discussion, we will like to discuss with you very specific aspects of MHI and how you will like each of the specific components to be designed to reflect the way you would like it to be. Specifically, we will discuss in detailed the services that should be covered by the MHI, the providers that should provide the services to those who will be insured, the contributions that you will like to make to the scheme and how you will like the scheme to be managed among others.*

**4.1 Purchasing** (**Benefit package: Service and cost coverage)**

**Preamble:** MHI covers various different types of health care services including services such as: ***(Moderator to read out the list of services from Table 1 and illustrate them to participants using local images).*** However, we recognize that in Malawi, there are already some services that you don’t normally have to pay for at certain health care facilities, thus they are supposed to be free of charge. But we don’t know if in practice you really do not pay for those services. Therefore, we want to find out from you, if a MHI system is supposed to be introduced within your district:

4.1.1. What kind of health services would you like to be covered by the scheme (included into the benefit package)? ***First, allow participants to list the services without probing, and then*** ***probe for other services using Table 1 as a guide)***

*For each service that will be mentioned, the moderator will ask*

*4.1.2.* Why would you like it be covered under the insurance scheme?”

4.1.3 Do you know whether that service you would like to be included in the MHI benefit package is already supposed to be free or not? If yes, then why do you want it to still be covered under the MHI scheme?

*4.1.4. For those services that will be probed for, the moderator will ask*, do you think (*name of service listed in Table 1*) should not be covered? Why?

**4.1.5. Preamble:** Due to limitation of the fund, most insurance schemes usually cover part of the cost of the health services in the benefit package and the client also pays part. It must be noted that the proportion of the cost that will be covered by the scheme will depend on the amount of money that will be contributed by each person. You mentioned **(*facilitator to restate all the services participants mentioned earlier)*** as the services you will like to be covered. Now, how will you like the cost for each of the services to be covered and why?

- *Probe, for levels of coverage between full 100% and partial coverage (if partial let them suggest proportions 25% and 100%*

**4.1.6.** As a group can you order all the services that you will like to be included in the MHI benefit package (*facilitator to restate the services*) according to the ones that are most important and hence should be covered first?

4.3.8. What criteria did you consider in ordering the services?

**4.2. Service provision under MHI**

4.2.1. Normally when you fall sick, where do you often go for health care? Why?

- Probe; for types of health care facilities: CHAM, private, public and traditional healers.

***4.2.2.*** What kind of health providers will you go to for health care if you have an insurance cover that will pay for your treatment? Why? Probe for: Public, Private, CHAM and traditional practioners)

4.2.3. How will you like the health providers who will provide the services to you under the insurance program to be assigned to you? And why?

Probe for the following:

- should be allocated to just one provider (public, or CHAM or Private)
- Should be allowed to go to any provider of their choices any time sick
- The insurance scheme should establish its own providers to specifically provide services to only the insured clients

**Collection of premiums and contributions**

- - 1. How would you like the contribution/premium (money) each person will be expected to make into the common basket (insurance fund), to be charged? Why? Probes:
- Per individual or household? Why?
- Sub-probe: If per household, how many people and who should comprise a household?
  - As a flat rate for everybody or according to people’s earnings: those who earn more pay more? What about those who are very poor and cannot pay at all?
- Sub-probe for only SACCO members: probe for as a percentage of SACCO savings or loans?
  - According to age: Adult and children?
    1. How often would you like the premium to be collected from you? Why?

Probe:

- - Monthly and annual premiums.
  - Sub-probe: If annual, probe for what time (s) or seasons should the premium be collected?
    1. You mentioned (moderator to list services mentioned by respondents they may like to be included in the MHI benefit package), how much do you think will be a reasonable amount that you would be willing to pay per month or per year to be enrolled into the scheme? Why?
- Probe for different amounts per individual and per household.
  - 1. Who should be responsible for paying this premium for the individual or the household that will be enrolled into the MHI? Why?
- probe for employer, individual, household head
  - 1. How will you like the premium to be collected from you? Probes:
  - Forms of premium payments: cash and kind
  - Means of collecting the premium/contributions: paying through community agents, paying directly to management staff of the fund, deduction from salary etc.
  - Possibility of payment by installment?
  - Additional probe for SACCO members: deduction from SACCO accounts?

**4.4. Pooling levels and management**

- - 1. How would you like the day-to-day management of the MHI, for example issues relating to the money that you will contribute into the common basket (fund) and how it will be used, to be handled? Why? Probes:
- By community elected representatives?
- By an NGO?
- By health care provider?
- Additional probe for SACCO: by SACCO management?
  - 1. Will you be willing to contribute into a MHI fund (the common basket) if it will be used to pay for the health care cost of you, your household and other members of this community (mentioned name of community)? Why?

Further probes:

- What about if the fund (common basket) will be used to pay for the health care cost of all members of the entire traditional authority (mentioned name of STA, TA, TOWN)?
- What about if the fund (common basket) will be used to pay for the health care cost of all members of the entire district (mentioned name of district)?
- Additional probe for only SACCO members: what about if only the members of the Bvumbwe SACCOs contribute to and benefit from the fund?

1. **Information communication channels under MHI**

If a MHI is launched in this district (name of district), what are the various ways by which information on the system can easily be delivered to you and other resident in the community? Probe:

- Community durbars?
- Radio/ TV programmes/newspapers
- Billboards and posters?
- Health care providers?
- Additional probes for SACCO: fellow SACCO members, loan group meetings and SACCO general Assembly)

1. After going through this whole discussion on MHI, can you now again as a group tell me what specific elements that you would like to be taken into consideration when designing a MHI product? (facilitator to summarize the key elements and allow groups to agree on the most important ones, 5 points should be ok)
2. Is there any other element concerning the introduction of MHI, that we have not discussed and you will like us to addressed?

**Thank you very much for your time**

1. **Key informant interview guide: Deriving attributes and attributes levels of MHI in Malawi: (providers of health services: administrative and clinical staff)**

***(Note: For attributes and levels derivation, go to part C and Part D)***

Part A: Basic information on respondents (to be printed out and filled by interviewer)

| Interview number |  |
| --- | --- |
| Date of interview |  |
| Name of district |  |
| Setting/Location of facility |  |
| Name of community where facility is located |  |
| Name of facility |  |
| Type of health facility (Public hospital, public clinic, Private or CHAM etc) |  |
| Profession of interviewee (nurse, doctor, hospital administrator/accountant) |  |
| Interviewee’s highest level of education |  |
| Duration of working in the health sector of Malawi |  |

**Introduction**

I am (Gilbert Abiiro). I am from Ghana, but currently working at the University of Heidelberg in Germany. The University of Heidelberg is jointly conducting this study with REACH TRUST, a research institution in Lilongwe. This interview that I wish to have with you today is part of a bigger study that is aimed at assessing the potentials of a proposed health insurance program in meeting the health care needs of people within this community or district. Some organizations are proposing to introduce this health insurance program for people within this area. In these interviews we are specifically interested in understanding how this proposed health insurance system can be designed to reflect the preferences of the residents of this community. As a health worker within this community/district, you interact with community members regularly and hence based on your experience as a health worker, we believe you will have a good understanding of the health care needs of the community members and their health seeking behavior. This is the reason why we have selected you for this interview. The interview will specifically cover the following issues:

- The health care needs of the people within this community/district and the problems they face in accessing the needed health care
- Your understanding of and experience with health insurance in general
- I will introduce to you the type of health insurance that is being proposed and I will like to get your opinion on the elements that should be considered important in designing such a system
- I will also like to know what you see as the important elements that will influence decisions of residents of this area to join or not to join such a program if it is present here in the community or district. The discussion will specifically cover topics including the contributions that people will be expected to make to the system, the benefits that they will expect to derive from it and how the system should be managed.
- I will also specifically ask you to identify and justify the kind of health services you and your clients may like to be included in the benefit package of this program

The interview will last not more than 1 hour.

We have obtained ethical clearance for this study from the University of Heidelberg and the Malawi National Health Sciences Research Committee.

Statement of informed consent: *(interviewer to read out from written informed consent form and obtain a signature from interviewee)*

***Start tape recording if consent is granted: (interviewer to switch recorder on)***

**Part B: Introductory questions**

1. **Community health care problems and needs**.
2. What do you think are the main problems that residents of these community or district (name) face in accessing the health care they need? Why?

**Probes:** *(probe only if the issue is not mentioned):*

- Cost of health care
- Availability of health providers and medical products
- Quality of health care

1. Do you think anything could be done to help overcome such problems? If so, what? And by whom?
2. **Awareness and understanding of health insurance**
   1. Have you ever heard about health insurance?

- Probe for knowledge on different types of insurance (for commercial and not-for profit) (If no go to 2.7 and then continue from 2.11)
  1. How did you first hear about health insurance?
- Probe; using names such as medical aid, sickness funds, mutual health Organizations.
  1. How would you describe the function of a health insurance?
- Also probe for common understanding of health insurance)
  1. Are you a member of any health insurance scheme in Malawi? (If no, why? And go to 2.7?
  2. What is the name of the health insurance scheme you belong to?
- Probe for whether it is private, employer based etc)
  1. Describe to me how the health insurance scheme you belong to operates?
  2. What kind of clients do you receive at this health facility?
- Probe for: different kinds, poor or rich, clients within Malawi or outside as well?
  1. Do you receive clients in your health facility that come with health insurance coverage? If no, go to 2.11
  2. What kind of health insurance schemes do those clients belong to?
- Probe for names and types?
  1. What kind of health care services do those insurance schemes pay for patients in your facility? Probe using list of services on table 1
- Also, probe for the proportion of cost paid by the insurance for the services
  1. Those patients who are not on health insurance, how do they normally pay for their health care services in your facility?
- Probe for different payment mechanisms such as out of pocket and free?
  1. What kind of health services do people normally pay out-of-pockets in this facility? Probe using list of services on table 1
  2. Are there any health services you think residents of this community need but they are not available at all?
- Probe for types of services and why they are not available.

2.14. Do you know the kind of health services that are supposed to be free in Malawi (covered under the EHP?) probe for list

2.15. Which of those services will you say people sometimes still have to pay for and why?

**Part C: Introduction to MHI**

*In the rest of the following sections, we are going to* discuss issues relating to one specific type of health insurance. *(If the interviewee is aware of health insurance: interviewer will read this to him/her, those schemes that currently exist in Malawi (restate names of schemes) are either private health insurance schemes or employer specific schemes*. *The private health insurance schemes are like business organizations and hence aimed at making profit).*

The type of health insurance that I am here to discuss with you about, is supposed to be a system that will be designed based on the interest of the people it intends to cover and will not be for profit. Such a system is called micro health insurance. In some places, this MHI is called community-based health insurance, micro insurance, medical aids (but not for profit), mutual health organisations, community health funds, sickness funds or rural health insurance.

MHI is a system whereby a group or community pools money together into a common fund through regular contributions which is used to cater for their health care needs. Within a MHI, everyone in a group (for example a MFI group) or a community (for example name of community) or within the entire district (name of district), whether rich or poor, healthy or sick, young or old, who is willing pays money (small amounts called insurance premiums) regularly into a health insurance fund which is used to pay for the health care cost of a defined service benefit package for those of them who will fall sick. Usually, the health insurance pays the health care providers for the services delivered to the insured client out of the insurance fund.

MHI systems exist in a number of communities in Ghana, Burkina Faso, Cameroon and some other countries in the world. In one district in Cameroon for instance, the people have established a MHI which is managed by elected representatives of the community. To become a member of the MHI, people pay a registration fee and an annual contribution (premium) into the common fund to cover their entire household members (usually only 4 people). Each member is given a membership identity card and any time the member falls sick, s/he goes to the hospital with his/her card for treatment and the insurance fund pays 75% of the total cost of the treatment for the member [1]. In Ghana, a similar system exists, but in the Ghanaian system the insurance pays for all the cost of the services provided. Each person also contributes to cover only him/herself alone but not the entire household. In Ghana also, children and the very poor in the community are not expected to contribute into the fund but they also enjoy the health services free of charge and the insurance pays for them. As a principle of health insurance, the contributors are usually not given a refund of the premium if they pay but do not fall sick at all and hence do not benefit from the fund. This is because their contribution is also used to treat those who will fall sick. Hence the members of MHI turn to support each other to pay for their health care bills and share each other’s risk. In both the Ghanaian and Cameroon examples, every member is expected to pay his/her contributions (premium) every year irrespective of whether the member benefited from the scheme in the previous year or not.

It must be noted that, how people contribute into this insurance fund, who benefits from this fund, the cost of what health care services should be paid from this fund, how these health care services will be provided and how the insurance system will be managed, are usually decided when designing the system. These are among the several things I will like to discuss with you today.

As health care providers, you are partners of this system because you will be expected to provide services to the insured. As stated earlier we believe that you will therefore have a better understanding of community behavior and preferences in relation to issues of health care. Hence, I would like you to give me your candid opinion on the following questions.

***3.0.*** **Understanding of the basic principles of MHI and their general opinions about MHI**

- 1. Do you think a system like this would provide advantages or disadvantages for your clients? How would you justify your answer?
  2. As a health care provider, do you think a system like this would provide advantages or disadvantages for you and your facility? Why?
  3. Do you think your clients would like to contribute to and benefit from the services of such a system? Why?

**Part D: Deriving attributes of MHI**

4.1. If you are asked to give your opinion on how this MHI can be set-up within this district in order to reflect the way the community would like it to be, what specific elements come to your mind spontaneously that you will consider very important in designing such a MHI? And why?

Probes: (*probe only if respondents is finding difficulties in answering the question)*

4.1. The premium and how it will be paid?

4.2. The type of health services that will be covered?

4.3. The providers that will be contracted? etc.

Also, probe for what community member members will consider important in deciding to join or not

4.2. As a health care provider, do you think the way you would personally like a MHI to be set-up within this area will be different from the way the community will like it to be? Justify your answer.

**Part D: Deriving attribute levels**

*I will now ask you more specific questions on your opinion about what community members will like in relation to how the money (premium) will be collected, pooled together, managed and used to purchase health services for them under a MHI.*

- 1. **Preamble:** Most health insurance schemes cover the following services in the benefit package: ***(Moderator to read out the list of services from Table 1).*** However, we recognize that in Malawi, there are already some services that patients don’t normally have to pay for at certain health care facilities, thus they are supposed to be free of charge (covered under EHP). So, if a MHI system will be introduced within this district ( mentioned name of the district):
- What kind of health services do you think people within this community who wish to join the MHI scheme, will like to be included into the insurance benefit package?” Please try to list services in order of importance.
- Why do you think they will like the listed services to be included in the benefit package? (Interviewer will restate each of the health services mentioned and the respondent will assign the reason to it)
- Are those services you mentioned already supposed to be free (covered under EHP)? If yes, then why do you think they will still want them to be covered under the MHI scheme?
- Probe further for other services*: those services that will be probed for, the moderator will ask*, do you think (*name of service listed in Table 1*) they would not like this (name) to be included in the benefit package? Why?
  1. **Preamble:** Sometimes, due to limitations of the fund, the MHI scheme may cover part of the cost of the services in the benefit package and the client also pays something small. It must be noted that the proportion of the cost that will be covered will depend on the amount of money that will be contributed by each person. You mentioned, **(*interviewer to restate all the services earlier)*** as the services they may like be covered. Do you think members of this community will accept to enroll into the scheme if it does not cover the full cost of each of the above services? Why?
- Probe for different coverage levels that may be acceptable
  1. In this community, when people fall sick, which kind of health care facilities do they normally go for health care? Why?
- Probe for public, CHAM, Private and traditional healer
  1. If the people from this community or district join a MHI scheme that will pay for the cost of their treatment, what kind of health care facilities/providers do you think they will like to visit when sick and why?
- Probe for public, private and CHAM
  1. How do you think your clients will like the contributions (premiums) that each will make to the MHI to be charged?

Probes:

- Per individual or per household? Why?
- Flat rate or according to peoples earnings? Why?
  1. You mentioned (Interviewer to list services mentioned by respondents first as health services that clients may like to be included in the MHI benefit package), how much do you think will be a reasonable amount that your community members or clients can pay per year to the insurance scheme? Why?

Probe: Do you think that paying per year or per annum is the best?

- 1. What forms of payment do you think your clients will like? And why?
- Probe for cash and kind
  1. What will you recommend as the most appropriate means of collecting the premium/contributions from your clients? Why?
  2. What do you think will be the most favorable time (s)/ season(s) of collecting this premium from your clients? Why?

5.10. How do you think your clients will like the insurance fund to be managed? Why? Probes:

- By community elected representatives?
- An NGO?
- Health care provider?

5.11. If a MHI is launched in this community, what are the various ways by which information on the system can easily be delivered to community members? Probes: through:

- Through the local leaders
- Community durbars/sensitisation?
- Radio/ TV
- Billboards and posters?
- Health care providers?
- Probe: about SACCO management

6. Is there any other element concerning the introduction of MHI that we have not discussed and you will like us to address?

**Thank you very much.**

Pictorial illustration of MHI used during FGDs^1^

[1]

**Reference**

1. Rösner H-J, Leppert G, Degens P, Ouedraogo L-M. Handbook of Micro Health Insurance in Africa. Berlin: LIT Verlag; 2012.
